# Extraluminal bronchial blocker placement using both nostrils for lung isolation in a patient with limited mouth opening: A CARE-compliant case report

**DOI:** 10.1097/MD.0000000000021521

**Published:** 2020-08-07

**Authors:** XianHe Zheng, ChangFeng Zhang, ShuMei Lian, ShuYun Liu, ZongMing Jiang

**Affiliations:** a Department of Anesthesia, Shaoxing University Affiliated First Hospital; b Department of Anesthesia, Shaoxing People's Hospital, Shaoxing 312000, Zhejiang Province, China.

**Keywords:** one lung ventilation, bronchial blockers, single lumen tube, nasal cavity, difficult airway

## Abstract

**Rationale:**

The establishment of lung isolation is often particularly challenging for the anesthesiologist in patients with difficult airway. Usually, orotracheal intubation with double lumen tube is the commonly used technique for achieving 1 lung anesthesia. Whereas, in patients with limited mouth opening and restricted cervical mobility, this technique becomes extremely difficult and hazardous. We report a case in which bronchial blocker placement was succeeded via both nostrils in a difficult airway due to restricted mouth opening.

**Patient concerns:**

A 50-year-old, non-smoking female with a painless mass in the left upper lobe. She had a 10-year history of ankylosing spondylitis and squamous cell carcinoma of the floor of the mouth after 5 operations 4 years previously.

**Diagnoses:**

Left upper lobe adenocarcinoma, ankylosing spondylitis and oral squamous cell carcinoma.

**Interventions:**

To achieve 1 lung anesthesia, both nostrils were used for extraluminal bronchial blocker placement.

**Outcomes:**

Initially, oral intubation was selected for establishing a patent airway but failed. Then switched to nasal canal for insertion, after several attempts, a conventional nasal intubation tube (internal diameter 6.0 mm) was placed via 1 nostril under topical anesthesia, with the aid of a flexible fiberoptic bronchoscope, and a bronchial blocker was advanced to the desired position via the other nostril.

**Lessons:**

In difficult airway with limited mouth opening and restricted cervical mobility, multidisciplinary experts participated discussion is a prerequisite for contemplating a scientific plan. Preoperative computed tomography scan and 3-dimensional computed tomography reconstruction would be helpful in detecting the narrowest part of airway conduit and determining a safe, reliable, and feasible airway program.

## 1 Introduction

Isolation of a lung and the 1 lung ventilation technique are commonly used during thoracic surgery. A double lumen endotracheal tube or a single lumen tube in conjunction with a bronchial blocker is generally used for attaining lung isolation via the orotracheal route.^[1–3]^In the difficult airway, orotracheal intubation using a single lumen tube with intraluminal or extraluminal placement of a bronchial blocker is often the preferred choice to achieve lung isolation. However, in patients with limited mouth opening due to maxillofacial disease, it is not easy to establish a patent airway through the oral route. In these circumstances, careful preoperative planning and multidisciplinary teamwork are essential to establish a patent airway and an uneventful outcome. Here, we report a case of a patient with limited mouth opening, who was intubated via 1 nostril, guided by fiberoptic bronchoscope (FOB), after which a bronchial blocker was introduced with the help of FOB through the other nostril. The patient agreed to publish the case report and signed the written informed consent. Shaoxing People's Hospital Ethics Committee waived the ethic censorship for the case presentation.

## 2 Case Presentation

The institutional ethical review was waived and informed consent was obtained from the patient. A 50-year-old, non-smoking female (height 155 cm and weight 50 kg) was transferred to our hospital after a painless mass was detected in the left upper lobe, on a routine thoracic computed tomography (CT) scan. It was diagnosed as adenocarcinoma by fine needle aspiration biopsy. She was scheduled for elective video-assisted lobectomy surgery. During the preoperative anesthetic assessment, we found that she had a 10-year history of ankylosing spondylitis and had undergone 5 operations 4 years previously, on her mandible and floor of the mouth, with radiotherapy, because of squamous cell carcinoma of the floor of the mouth. More significantly, due to postoperative adhesions, scarring and skin induration, she had limited mouth opening and reduced masticatory function, requiring a predominantly liquid diet. Physical examination revealed normal breathing sounds and no neurological abnormalities. Cardiac examination was normal, with a cuff blood pressure of 130/80 mm Hg and a heart rate of 74 beats per minute. Preanesthetic assessment showed a maximum mouth opening of 8 mm (from upper incisal edge to lower incisal edge), a stiff mandibular joint, 5.5 cm thyromental distance, modified Mallampati airway score of grade IV, and head-cervical mobility below 60°. The patient was grade III on the American Society of Anesthesiologists physical status grading, and grade A according to the American Heart Association system. Laboratory examinations, such as routine blood tests, blood coagulation function and hepatic function were within the normal ranges. A CT scan of the head and neck showed loss of cervical physiological flexion, intervertebral fusion, and rigidity after ankylosing spondylitis and partial oral muscle contracture after surgery. During the preoperative visit, the patient expressed her willingness for surgery and firmly refused a tracheotomy under any circumstances. Based on this preoperative assessment, a comprehensive plan was developed by our multidisciplinary team aimed at establishing a patent airway without putting the patient at risk.

Preoperatively, the patient fasted for 6 hours for solid food and 2 hours for clear fluid. She was premedicated with 150 mg ranitidine orally and 10 mg morphine intramuscularly. On arrival at the theatre, a 16-gauge cannula was inserted in a peripheral vein and electrocardiography, invasive radial artery catheterization, and pulse oximetry monitoring were initiated. Twenty milliliters of 2% lidocaine were added to a nebulizer and inhaled via a face mask for 20 minutes before anesthesia. A loading dose of dexmedetomidine 1.0 μg/kg was injected (infused over 10 minutes) followed by nasally-dripped ephedrine 6 mg and 3 mL of 4% lidocaine. Thyrocricocentesis was then conducted followed by 5 mL of 2% lidocaine spray for topical anesthesia, and finally 0.04 mg/kg midazolam was intravenously administered. The procedure began while maintaining a Ramsay sedation score between 2 and 3. According to the preoperative plan, we initially attempted to intubate using a conventional internal diameter (ID) 7.5 mm tube under FOB guidance. Contrary to our expectations, it encountered resistance because of the limited mouth opening. Smaller tubes, ID 7.0 mm or 6.5 mm were attempted but without success because of the poor accessibility. After a short discussion, our team members decided to use a nasal approach for establishing the airway. We tried 3 times with the aid of FOB, using an ID 7.0 mm and 6.5 mm single lumen tube via the right nostril. Unexpectedly, the tube would not pass, kinking at the middle turbinate; therefore, we had no choice but to remove it forcibly. Unfortunately, mucosal laceration during extubation caused epistaxis. At this time, the patient became agitated, disappointed by our several failed attempts. We immediately discontinued the procedure, used compression for hemostasis and talked to the patient. After a conversation with the patient, she agreed to cooperate while we made another attempt. An ID 6.0 mm single lumen tube (Cook Medical, Bloomington, Indiana) was advanced into the trachea through the right nostril with the aid of FOB. With the airway secured, the patient was immediately administered midazolam 0.05 mg/kg, sufentanil 0.3 μg/kg, cis-atracurium 0.2 mg/kg, and propofol 1.5 mg/kg. A bronchial blocker (DLT-3003, Zhejiang Haisheng Medical, China) was inserted to the desired position via the left nostril with the help of the FOB. At the proximal end of the blocker was a rotating wheel to enable directional control of the flexible blocker tip. When resistance was encountered during blocker placement, the tracheal tube cuff was deflated and then the tube was withdrawn until the cuff balloon could be seen at the lower margin of the vocal cords. Finally, after 3 hours of effort to control the airways, surgery was performed uneventfully. The tube was removed when the patient was fully awake. She recovered progressively and was discharged after 7 days in hospital.

## 3 Discussion

One lung ventilation is required during most thoracic surgeries to provide a clear surgical field and good surgical access. Establishing 1 lung ventilation is often challenging for anesthesiologists, especially in patients with difficult airways. In the clinical context of difficult airway, several instruments can be used for achieving lung isolation, for instance airway exchange catheters, intubation-tool assistance devices such as GlideScope (Verathon Inc., Bothell, WA), the gum elastic bougie, and the Trachlight (Laerdal Medical, Armonk, NY) for insertion of a double lumen tube.^[4]^ In patients with difficult airway, bronchial intubation intentionally using a single lumen tube is often the first choice, despite the likely need for repeated withdrawal and advancement intraoperatively.^[5–7]^ However, these commonly used instruments cannot be used in patients with limited mouth opening and joint rigidity. Under this situation, a bronchial blocker in conjunction with single lumen-tube is often indicated for 1 lung anesthesia and for selective lobar collapse due to bronchial blockers associate with less incidence and severity of airway injury, easier to enter in the narrow space. Notably, use of bronchial blocker has many disadvantages, including unable to aspirate the secretions, malfunction of the blocker, longer time to position for poor knowledge of endoscopic bronchial anatomy and occasional enclosed the blocker into the surgical staple line during lobectomy, and so on^[1]^ Despite the disadvantages, the superiority of bronchial blocker in achieving lung isolation renders it is a good selection in patients with restricted mouth opening. Except for instruments selection and consideration, detailed preoperative preparation with flexible strategies for overcoming unexpected problems, are the basic components for ensuring patient safety and smoothly establishing 1 lung anesthesia.

We organized a multidisciplinary team of experts and drew up a detailed plan for this case. The procedural goals were as follows. Securing the airway was the first priority and lung isolation was a secondary concern, because of the difficult airway.^[7]^ Second, orally awake intubation using a single lumen tube was the first choice because a large-diameter tube would be advantageous for thoracic anesthesia. Third, the patency of the nostrils must be assessed and they should be considered as an alternative approach for intubation. Fourth, different sized FOB from outer diameter 2.2 mm to 3.8 mm and related instruments for intubation must be available. Finally, if all attempts at intubation and lung isolation failed, the surgery would be canceled. In the present case, the pre-existing physiological conditions including the maximum interincisor gap of 8.0 mm, oral cavity scarring and adhesion formation, and cervical mobility restriction due to ankylosing spondylitis and radiotherapy treatments, increased the difficulty of establishing a patent airway. Initially, we expected that the maximum interincisor distance would be wider once the muscles relaxed under sedation. Therefore, we intended to intubate orally with an ID 7.5 mm single lumen tube. Conversely, the interincisor distance became narrower, due to involuntary muscle contraction in conjunction with the stiff mandibular joint. Consequently, we attempted intubation with the smaller ID 6.5 mm tube, but failed because the smaller tube still had an external diameter larger than the interincisor distance. After several failed attempts, we switched to the right nostril and attempted to intubate with an ID 7.5 mm single lumen tube. Although this failed, an ID 6.0 mm tube was successfully passed through the nasal cavity. However, 1 lung ventilation could not be achieved because the ID 6.0 mm tube was not adequately long to access the right bronchus. Moreover, there was inadequate space next to the ID 6.0 mm tube to permit the entry of a bronchial blocker and navigate it under FOB guidance. Therefore, it was decided to use the left nostril to place a bronchial blocker beside the intubation tube, to collapse the lung.

Due to the lack of a preoperative, 3-dimensional head and neck CT reconstruction, the precise pathologic changes of the bone and soft tissue were not known, and importantly the narrowest point in the nasal cavity was not identified, which might be the cardinal reason for our failed nasotracheal intubation and the subsequent epistaxis. Inevitably, this process was time consuming and painful for the patient. Accurate measurement of the narrowest point in the nasal cavity would also have been helpful for the accurate selection of the tube size. In addition, we did not consider whether the tube was adequately long to achieve lung isolation via the nasotracheal route. In this case, a preoperatively-prepared, extra-long, single-lumen endotracheal tube or endobronchial tube, known as a “supertube”,^[5]^ might have been helpful, even eliminating the need for an independent bronchial blocker. The outer diameter of a conventional tube is often narrower than wire-reinforced endotracheal tube or Fuji silbroncho double lumen endotracheal tube with the same inner diameter.^[1]^ Moreover, a large ID tube is often preferred in thoracic anesthesia.^[8]^ Therefore, in our case, we selected a conventional tube for intubation.

## 4 Conclusion

Based on previous literatures, from this case we conclude the following for future considerations. Most important is avoiding a scenario where a difficult airway becomes an emergency. Securing the airway is of paramount importance in an anticipated difficult airway. Second, thoughtful preoperative preparations and formulating a comprehensive plan are prerequisites to ensure patient safety and successful airway establishment. Finally, in patients with limited mouth opening and restricted cervical mobility, a preoperative CT scan and 3-dimensional CT reconstruction would be helpful in determining a safe, reliable, and feasible airway management program.

## Author contributions

Xianhe Zheng and Changfeng Zhang prepared and wrote the manuscript, Shumei Lian and Shuyun Liu provided the critical review. Zomgming Jiang conceived the study and prepared the material.

## References

[1] Collins SR Titus BJ Campos JH Lung isolation in the patient with a difficult airway. Anesth Analg 2018;126:1968–78. 29189274 10.1213/ANE.0000000000002637

[2] Campos JH Lung isolation techniques for patients with difficult airway. Curr Opin Anaesthesiol 2010;23:12–7. 19752725 10.1097/ACO.0b013e328331e8a7

[3] Purohit A Bhargava S Mangal V Lung isolation, one-lung ventilation and hypoxaemia during lung isolation. Indian J Anaesth 2015;59:606–17. 26556920 10.4103/0019-5049.165855 PMC4613408

[4] Brodsky JB Lung separation and the difficult airway. Br J Anaesth 2009;103 (s1):i66–75. 20007992 10.1093/bja/aep262

[5] McLaurin S Whitener GB Steinburg T A unique strategy for lung isolation during tracheobronchoplasty. J Cardiothorac Vasc Anesth 2017;31:731–7. 28159522 10.1053/j.jvca.2016.11.027

[6] Liu Z Yang X Jia Q One-lung ventilation in a patient with a large mass on the glottis: a case report. Medicine (Baltimore) 2018;97:e12237. 30200151 10.1097/MD.0000000000012237 PMC6133415

[7] Müller SHS Defosse##JM Gerbershagen MU Difficult airway management in thoracic anaesthesia. Anasthesiol Intensivmed Notfallmed Schmerzther 2018;53:187–97. 29554711 10.1055/s-0043-114679

[8] Okuda I Yamase H Ogawa S Imaging aid for thoracic surgery: multidetector-row computed tomography evaluation of the tracheobronchial structure and bronchial tube selection for one-lung anesthesia. Gen Thorac Cardiovasc Surg 2009;57:369–75. 19597927 10.1007/s11748-008-0386-9

